# Life stories: Unraveling the academic configuration of a multifaceted and multidisciplinary field of knowledge

**DOI:** 10.3389/fpsyg.2022.960666

**Published:** 2022-11-17

**Authors:** Rocío López-Montero, Carmen García-Navarro, Antonio Delgado-Baena, Rocío Vela-Jiménez, Antonio Sianes

**Affiliations:** Research Institute on Policies for Social Transformation, Universidad Loyola Andalucía, Córdoba, Spain

**Keywords:** life stories, biographical method, bibliometric analysis, scientific production, VOSviewer

## Abstract

In the field of qualitative research, life stories are consolidated as one of the most important techniques within the biographical method. However, due to the multiplicity of techniques covered by this method and the disciplines in which it is applied, the contributions and scope of life histories do not present a clear delimitation. By contrast, a considerable conceptual confusion persists and the transfer of its production remains very narrow. In this sense, this article aims to clarify the field of knowledge generated through life stories. To this end, it innovatively applies the bibliometric method. Making use of performance analysis and scientific mapping through the VosViewer application, it studies a body of 2670 articles indexed in the Web of Science. The results show how knowledge transferred from psychology through its major schools of thought occupies a central place. This leaves in a secondary position the knowledge produced by other disciplines such as sociology or anthropology, which is not transferred in the main forums of scientific impact. In this way, the conclusion points to the need to open up new lines of research to find out the differences between the different techniques and disciplines when applying this methodology.

## Introduction

### The biographical method

One of the most commonly used approaches in qualitative research is the biographical method (Rubio and Varas, 2004). However, and despite its high contribution to the scientific field over time, it has not been exempt from limitations, which have hindered the development of its full potential (Peacock and Holland, 1993) to these days. It is composed of a wide variety of vaguely defined techniques (Pujadas, 2002), which leads to conceptual confusion. Additionally, there are different orientations depending on the disciplines that use it (Bertaux, 1980), translating into divergent and disjointed results.

In this sense, it is necessary to reach a deep understanding to have an overview of the performance of this method, explain the key concepts, differentiate closely related or similar concepts, determine the characteristics that define different techniques as well as the traits that differentiate the use of such techniques by the different disciplines. This involves identifying the main research streams, emerging and underexplored areas, as well as knowledge gaps and lacunae. This knowledge will allow us to guide academia and to identify the necessary methodology for each investigation, while at the same time suggesting theoretical, contextual, and methodological opportunities and solutions to advance knowledge in this field.

Given the dispersion of the biographical method and to define a systematic approach to its use, we consider pertinent to focus the analysis on a particular technique rather than on the method as a whole: life stories.

### Life stories

The birth of the life story technique is related to the origin of the biographical method, in its beginnings the main tool of this method was conceptualized with the term life histories (Rubio and Varas, 2004). However, the term life stories soon emerged (Pujadas, 2002). Both terms coexisted during the first years of their existence and to a certain degree there is still an indiscriminate use of both terms interchangeably (Cordero, 2012).

The present study follows the trend that supports the term life story instead of life history when addressing research in the field of social sciences. First, there is no clear delimitation of the term life history with respect to its use in natural sciences (Nettle and Frankenhuis, 2019). Therefore, we consider that productions that have been developed around this term are not always related to the object of study. Second, the term life story, from its beginnings, has been oriented to the investigation of human experience (Cornejo et al., 2008), which is consistent with the focus of this research. However, we do look at the singular (life story) as well as the plural (life stories) of the word story because different authors use the terms differently to refer to this technique depending on the number of stories studied.

Once the field that will be the object of study has been delimited, it is necessary to identify divergences in the literature with regard to the use of the technique within different disciplines: anthropology and sociology, on the one hand, and psychology, on the other hand, from which two different visions of life stories have been developed (Peacock and Holland, 1993). The former disciplines conceive the technique as a tool for the collection of ethnographic material. Scientific works in these disciplines have been more oriented toward uncovering the bases of functioning of sociocultural systems than toward the analysis and understanding of individual trajectories (Hernández-Hernández et al., 2020). From psychology, the life story is conceived mainly as a vision of the subjective experience of the narrator (Peacock and Holland, 1993). In this subjectivist approach, the life story is understood as an expression or projection of the psychological dispositions and dynamics of the subject; therefore, it is used as a window to the psyche (Peacock and Holland, 1993).

This perceived differentiation in the disciplinary use of the technique is the starting point of this study, as it highlights the need to understand the production of knowledge generated with its use to explain if the characteristics differ or converge depending on the perspective from which this concept is projected.

Systematic literature reviews are among the most useful vehicles for advancing knowledge and promoting research (Elsbach and van Knippenberg, 2020). These can take various forms (Paul et al., 2021) Among them, we consider that the most appropriate methodological approach for conducting this study is bibliometric analysis.

### Bibliometric analysis applied to life stories

The continuous increase in scientific production has made bibliometric studies increasingly important (Madrid Martín et al., 2017). The ability to handle large volumes of scientific data and produce a high research impact (Donthu et al., 2021) has led to them being widely distributed across all disciplines (Serrano et al., 2019). And become increasingly prolific in high quality academic forums (Paul et al., 2021; Mukherjee et al., 2022). Furthermore, unlike other types of reviews, bibliometric reviews are objective and not prone to error because they are based on automated quantitative data and tools (Lim et al., 2022; Mukherjee et al., 2022).

Despite the huge scientific output on life stories and the need to establish reliable knowledge, research on life stories thus far has been unrelated to the application of this method. Addressing this work is therefore indispensable today. Bibliometric research presents unique opportunities to contribute to theory and practice (Mukherjee et al., 2022). Thus, following Lim et al. (2022) we could say that reviewing the literature on life stories is a necessary, important, relevant, and urgent task.

It is necessary to make a general assessment of this field of knowledge with the aim of reaching an in-depth knowledge of the production disseminated through life stories. This effort will allow us to go deeper into the definition of this technique, as well as to establish the idiosyncrasies of the work developed by each of the disciplines from which it is applied. In turn, bibliometric reviews allow scholars to gain an up-to-date and comprehensive understanding of the state of the field (Sianes, 2021) which will help them to better position their future research in terms of which technique to use, as well as exactly which of the many existing streams they wish to expand. Our last aim it enable future studies to compare the production developed by the other techniques of the biographical method in order to establish, in this way, the boundaries of each of them to avoid the existing conceptual confusion.

Furthermore, reviewing the literature in this field becomes even more relevant when the use of life stories not only has a long tradition, but continues to be widely practiced today. This makes it urgent to establish firm and reliable boundaries, as the production of knowledge generated through this technique continues to grow without clear trade-offs in its use. Continued reliance on incomplete and outdated knowledge can be detrimental to addressing current problems and their overall progress (Lim et al., 2022). Thus, the originality of our study lies both in the methodology used and in the results obtained after the application of the methodology. The application of this method allows an intensive review of the academic literature on life stories, shedding light and deepening the state of the art of this object of study (Rey-Martí et al., 2016).

To clearly report the work carried out, the rest of the article is organized as follows. In section “Materials and methods,” we present the method for the bibliometric analysis, and we detail the necessary materials for the application of this method. In section “Results,” we present the main results obtained after applying the different techniques, and we discuss the main findings as well as their implications for academia. In section “Discussion,” we present the conclusions. We point out the need to open new lines of research, and we close the study with the references consulted.

## Materials and methods

### Bibliometric analysis

The origin of bibliometrics can be dated to the beginning of the 20th century (Godin, 2006), but it was not until the 1960s that key methodological developments occurred (Pritchard, 1969). Since then, it has gained immense popularity due, on the one hand, to databases that facilitate the acquisition of large volumes of scientific data and, on the other hand, to the ubiquity and usefulness of bibliometric software to evaluate them (Donthu et al., 2021).

Large bibliographic datasets make classical review methods such as systematic review or meta-analysis (Donthu et al., 2021) cumbersome and impractical (Ramos-Rodríguez and Ruíz-Navarro, 2004). In contrast, bibliometric analysis is designed to handle large volumes of data, which shows its suitability when working with 1,000 of articles.

Bibliometric analysis allows for the study of the publication patterns of scientific production in a given discipline to quantitatively evaluate, through statistical calculations, said production (Koseoglu et al., 2016). Bibliometry, as a scientific discipline, is based on the search for statistically regular behaviors in the elements related to the production and consumption of scientific information over time (Ardanuy, 2012).

Through this evaluation, it is possible to identify aspects such as the works on which the analyzed matter is based (Jamal et al., 2008); the academics who generate the production (Schmidgall et al., 2013); the journals where the information is transferred (Svensson et al., 2009); and collaboration between researchers (Köseoglu et al., 2015). In the specific case of life stories, the lack of knowledge of these data provided the inspiration and purpose for this study.

Bibliometric research generally involves two main categories of analytical techniques, performance analysis and scientific mapping (Mukherjee et al., 2022). That is what Benckendorff and Zehrer (2013) call evaluative techniques and relational techniques.

Performance analysis is the hallmark of the studies (Donthu et al., 2021). Most frequently developed in traditional bibliometric studies (Koseoglu et al., 2016). Evaluation techniques represent the first level of complexity of bibliometric methods (Serrano et al., 2019). They focus on the impact of academic studies that evaluate performance through productivity measures, impact metrics and hybrid metrics (Sainaghi et al., 2018). These measures allow reaching global explanations of observed phenomena through the formulation and verification of compliance with bibliometric laws (Ardanuy, 2012). In this study, evaluative techniques are applied through three main laws, i.e., Price’s law, Lotka’s law, and Bradford’s law, to understand the level of scientific production and the productivity of the authors.

Scientific mapping examines the structural connections and interactions between research constituents (Cobo et al., 2011). Relational techniques, although less explored in bibliometric analyses, are more interesting from the scientific point of view because they involve a higher level of complexity with respect to the units of information and analysis (Serrano et al., 2019). These techniques allow us to obtain hidden relationships between topics, authors, and documents (Van Eck and Waltman, 2020), revealing the intellectual structure of a research field. These scientific maping analyses can help enrich the descriptive insights provided by productivity analysis (Lim et al., 2022) by pointing out gaps and social dominance or hidden biases in the field, as well as the implications of research for the development of theory and practice (Mukherjee et al., 2022).

In the present study, these techniques are used to assess the relationships between published research (Figueroa-Domecq et al., 2015) by implementing a network analysis that allows a representation of the co-occurrence of keywords as well as the co-citation of authors and documents. The latter are explored with the help of visualization techniques provided by tools such as VOSviewer, free software for creating, visualizing, and exploring maps based on network data (Van Eck and Waltman, 2020). The application of Vosviewer software allows though a clustering algorithm a deeper analysis (Waltman et al., 2010).

### Data collection

Preparing a protocol is essential to ensure careful planning, anticipating problems, reducing arbitrariness, promoting accountability, and maintaining the integrity of the research (Paul et al., 2021). In our case, we have used the Scientific Procedures and Rationale for Systematic Literature Reviews, or SPAR-4-SLR based on three stages.

First, once the research domain has been identified, it is necessary to follow a series of steps to acquire the body of literature. We start by with the choice of an adequate database. For this study, the online database of the Web of Science (WoS) is the most relevant to achieve our aim because it allows access to source documents in our area of specialization (Mora et al., 2019) and contains data on results, dissemination, collaboration, and impact (Albort-Morant and Ribeiro-Soriano, 2016).

In addition, the data contained in the documents and indexed in the WoS have gone through a peer review process, guaranteeing “certified knowledge” (Ramos-Rodríguez and Ruíz-Navarro, 2004). Therefore, the search strategy included information pertaining to the journals classified in the Social Sciences Citation Index (SSCI) and the Science Citation Index extended (SCI-E) because both indexes contain journals in which life stories are published.

To extract scientific production from the WoS, a search vector is used. Taking into account these premises, the bibliographic sources were extracted by executing the following query: TS = (“life story” OR “life stories”). The search was not restricted to any particular language to avoid bias in the origin of the production.

Data collection was carried out on 22 September 2021. A total of 2,670 source documents were extracted from the database. The sample size corresponds to all publications related to the field of life stories located in the WoS. Regarding the time horizon, the analysis covers a period of almost a century because the sampling of extracted sources covers 1927–2021.

Once the data has been acquired, a second phase of data organization and purification takes place. It is necessary to take into account the existence of certain limitations in any bibliometric analysis. When using the information reported from science and technology information systems, it is common for there to be errors in the dataset extracted (Adam, 2002). Herein, manual verification was performed (Panori et al., 2019) in which some minor errors were corrected, not allowing the database to rectify the nomenclature when different ways of referencing the same author were identified. However, although it is necessary to consider this detail when interpreting the results of this study, previous studies have shown that the lack of accuracy in these terms does not affect the establishment of general patterns (Rodríguez Gutiérrez et al., 2017).

Finally, the data obtained is evaluated and analyzed below.

## Results

### Evaluation techniques

#### Law of exponential growth of science

Proposed by De Solla Price (1956), this law identifies behavioral patterns over time by distributing the frequency of publication per year to observe behavior by applying statistical adjustment functions (Kastrin and Hristovski, 2021; Delgado-Baena et al., 2022). This law postulates that the growth of scientific information is exponential and occurs at such a rapid rate that every 10–15 years, global information doubles. In addition, the author notes that over time, scientific production goes through the following stages: precursors–first publications in a research field; exponential growth–the field becomes a research front; and linear growth–growth slows and the primary purpose of publications is to review and archive knowledge (Ardanuy, 2012). However, each discipline undergoes its own evolution; therefore, it is necessary to assess the evolution of life stories.

**FIGURE 1:**
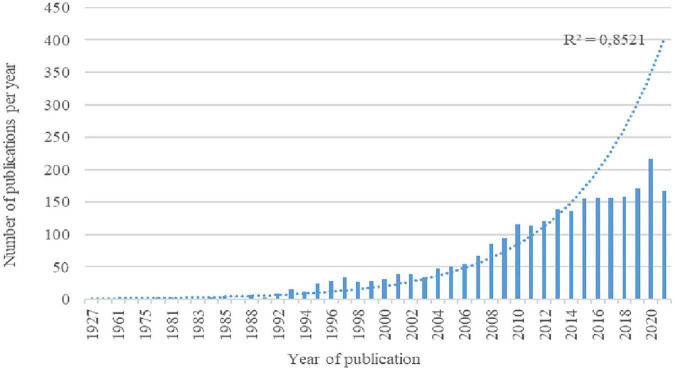
Number of documents published per year.

Figure 1 illustrates how the life story technique has been used since the 1920s, a fact that coincides with the date attributed to the birth of the biographical method (Pujadas, 2002). In this sense, the body of knowledge created through life stories has a trajectory duration of almost a century.

If we observe the trajectory of the publications throughout this time, *a priori*, three differentiated periods are observed.

The initial period spans from the late 1920s to the 1990s. In this phase, the pioneers of the discipline write the first articles that include this technique. In these early years, production was relatively low, especially that indexed in the most prestigious academic journals.

The second phase was when scientific production increased exponentially in the period from 1993 to 2013. This duplicity in production indicates that life stories became a technique commonly used by researchers during these 20 years.

The third phase includes 2014 to the present. In this last stage, there is linear growth, indicating that production stabilizes. However, the rebound observed in 2020 encourages exploration in the future if the use of the technique has reached a ceiling or saturation limit or if there is no growth in future years.

At a general level, the scientific information produced through life stories follows a normalized growth trend over time. The logistical function indicates that this field of knowledge has reached its maturity, strictly complying with Price’s law, which allows affirmation that life stories is a consolidated technique within the biographical method (Cordero, 2012).

#### Lotka’s productivity law

**FIGURE 2:**
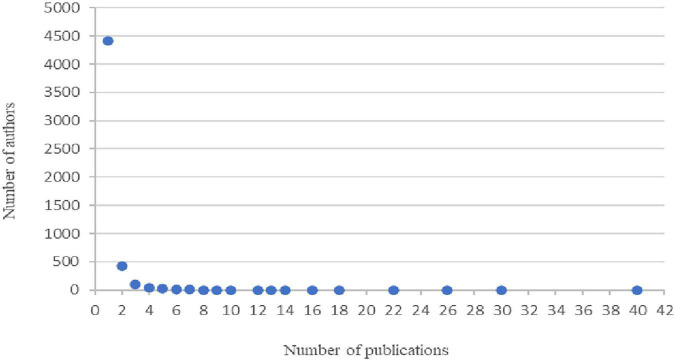
Authors and number of published documents.

This law, proposed by Lotka (1926), addresses the quantitative relationship between authors and their productivity in a given field over a period of time (Ardanuy, 2012). For authors, this discrete probability distribution is expected to be uneven, with most of the articles produced by a small number of very productive authors. The number of authors who contribute to a particular number of works is inversely proportional to the number of works contributed (Kushairi and Ahmi, 2021; Delgado-Baena et al., 2022). Productivity in life stories is shown in Figure 2.

Figure 2 shows how a small portion of authors produce most of the articles published on this topic, with a high percentage having low production. Of the 5,048 authors who have published using life stories, the maximum number of publications is held by a single author with 40 published articles. In contrast, 87% of the authors have contributed with a single publication. As in the majority of consolidated fields of knowledge, only a small number of authors are responsible for the majority of scientific works (González et al., 1997). Lotka’s law is also fulfilled.

Compliance with this law allows the most prolific researchers in the field to delimit the object of study. Table 1 provides the 10 main authors who are publishing life stories, as well as the field within which they do so. The selection of 10 authors as the threshold was made following the procedure reported in previously published bibliometric studies (Galvez, 2018; Sweileh, 2018).

**TABLE 1:** List of the most prolific authors.

Position	Most prolific authors	Discipline	Work area
1	Dan P. McAdams	Psychology	Personality
2	Dorthe Berntsen	Psychology	Memory
3	Dorthe Kirkegaard Thomsen	Psychology	Memory
4	Tilmann Habermas	Psychology	Autobiografical narrative
5	Kate C. McLean	Psychology	Personality
6	Robyn Fivush	Psychology	Autobiografical narrative and memory
7	William Dunlop	Psychology	Psychology social and personality
8	Jonathan M. Adler	Psychology	Personality
9	Monisha Pasupathi	Psychology	Autobiográfical narrative
10	Susan Bluck	Psychology	Memory

As seen in Table 1, the main producers of knowledge in the field of life stories are in the field of psychology, mainly personality, and memory. This finding confirms that the production of knowledge through life stories occurs mainly through psychology because the bulk of publications are attributed to this specialty as are the main reference authors. This fact corroborates the division of the psychological approach into the subjectivist and ethnographic approaches noted by Peacock and Holland (1993) and the predominance of the former over the latter. The invisibility of authors who work within sociology or anthropology points to the lack of transference of the works that are developed from these disciplines.

#### Law of dispersion in scientific bibliography

This law, proposed by Bradford (1934), estimates the exponential decrease in performance by broadening the search of references in journals. There exist core journals that address this topic in particular (Bradford’s core), the journals with more articles and citations are considered the core journals (Borgohain et al., 2021), and several groups that contain approximately the same number of articles as the core. However, recent authors argue that the effort to determine the exact Bradford zones is of no practical interest (Ardanuy, 2012) other than its descriptive aspect to deepen in what type of journals belong to the nuclear zone. To find this nucleus, it is necessary to arrange the journals in decreasing order of frequencies of articles on life stories and deepen knowledge of the sources where these studies are published. The results are shown in Table 2.

**TABLE 2:** Journals belonging to the Bradford nucleus.

Journal	Number of publications	WoS category
Memory	60	Experimental psychology
Journal of personality	46	Social psychology
Narrative inquiry	36	Communication/Linguistics
Qualitative inquiry	35	Interdisciplinary social sciences
Frontiers in psychology	30	Multidisciplinary psychology
Qualitative health research	27	Interdisciplinary social sciences/Biomedical social sciences
Applied cognitive psychology	25	Information sciences and librarianship/Social sciences, biomedical/Social sciences, interdisciplinary
Journal of aging studies	25	Gerontology
Women’s studies international forum	22	Studies of women
Developmental psychology	20	Evolutionary psychology

Table 2 shows that there is no clear leading journal where most articles on life stories are published. The literature is dispersed in a variety of sources belonging to this Bradford nucleus. However, when addressing the categories assigned by the WoS to the sources, the weight of the psychological perspective re-emerges. This, together with the prevalence of the biomedical social sciences category, indicates that more than half of the sources give priority to those articles produced from the most experimental or scientific social sciences, leaving production pertaining to collective social research dedicated to minorities, as pointed out by Bertaux (1980).

### Relational techniques

#### Proximity analysis

**FIGURE 3:**
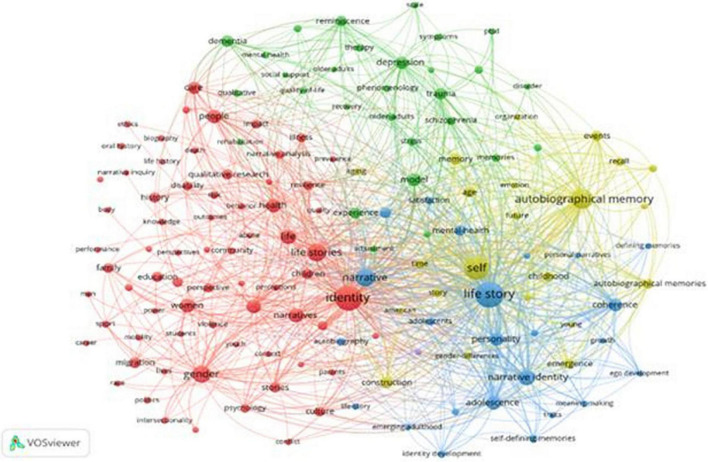
Keyword co-occurrence analysis.

Keyword co-occurrence analysis is the only bibliometric technique that uses the word as the unit of analysis (Donthu et al., 2021). It examines the actual content of the publication itself by assuming that words that appear frequently together have a thematic relationship with each other (Donthu et al., 2021). The co-occurrence analysis of keywords reflects the semantic structure of the research field under study. It is the most commonly used technique to identify relationships between the main research topics in a given scientific discipline (Muñoz-Leiva et al., 2012). Through this technique, two main results are obtained: the grouping of topics in clusters on the basis of their disciplinary proximity (Table 3) and the centrality of each topic within the literature as a whole (Figure 3; Sianes, 2021).

**TABLE 3:** Clustering of keyword.

Term	Occurrence	Term	Occurrence
**Personality and narrative identity**		**Autobiographical memory**	
Life story	365	Self	302
Narrative	170	Autobiographical memory	219
Narrative identity	122	Construction	76
Adolescence	95	Memory	71
Personality	92	Events	60
Coherence	87	Age autobiographical	58
Adults	61	Autobiographical memories	55
Adolescents	36	Childhood	52
Self-defining memories	34	Emergence	52
Autobiography	33	Time	48

**Mental health**		**Society and culture**	

Model	91	Identity	334
Depression	81	Life stories	165
Dementia	70	Gender	153
Trauma	69	Life	119
Experience	63	Narratives	109
Reminiscence	57	Women	105
Mental health	56	Health	101
Schizophrenia	39	Experiences	96
Stress	39	People	95
Therapy	37	Care	78

Figure 3 shows the existence of four large areas of research within the field of life stories. In Table 3, each of these four areas is defined as a function of the clustered key words.

##### Blue cluster: Personality and narrative identity

This cluster includes the most used concept in the entire body of literature: the life story. It is significant that the word most used refers to the technique in the singular, indicating a tendency toward the use of a single life story, displacing the need to reach a saturation point that validates the technique from disciplines such as sociology or anthropology (Bertaux-Wiame, 1980). Around this term are grouped words related to the development of personality and narrative identity. Likewise, there are terms related to adolescence and adulthood, interesting given that the transition from adolescence to adulthood is a critical period in the development of personality (Lüdtke et al., 2011).

##### Yellow cluster: Autobiographical memory

This cluster groups terms focused on the development of memory. The concept of oneself (self) as well as autobiographies (autobiographical) clearly shows the subjective orientation of life stories to which Peacock and Holland (1993) alluded. These works are not oriented to processes of collective memory. The prevalence of terms that refer to a moment of life, such as childhood or age, show the importance of temporal factors in the study of this topic (Bartolomé et al., 1996). Age is a widely studied variable because memory capacity in the early stages of life increases, while it decreases considerably in old age; therefore, it is a determining variable in studies developed in this field of research.

##### Green cluster: Mental health

This cluster includes, almost exclusively, concepts related to mental health, resulting in a very cohesive body of literature that represents a subset within psychology. The terms included in this cluster are related to the most clinical area of this discipline; the most prevalent keywords in the publications grouped in this cluster reflect research focused on the life stories of people with some disorder.

##### Red cluster: Culture and society

The most central word in this cluster is identity. Combining the results in Table 3 with those in Figure 3, the word life stories is nearby, which shows that the studies that nourish this cluster work with various life stories. This cluster links life stories to a series of specific areas, such as studies on women, homosexuality, old age, experiences of war, alienation at work, the world of drugs and prisons, or prostitution (Pujadas, 2002). All the words that this cluster encompasses indicate that this area of research includes both the work of social psychologists and scientists from disciplines such as sociology and anthropology.

In general, Figure 3 shows a very connected and dense network, where the clusters are very linked, indicating that the relationship between the different thematic areas is mature. In the first three clusters described, the link to psychology is very clear. The words that form them represent the works that are developed to access knowledge of the dynamics of the human psyche. Specifically, in the first two, the influence of evolutionary psychology is observed; studies assess these aspects from the perspective of vital development (Köber and Habermas, 2017; Camia and Habermas, 2020). The terms that are grouped in the last cluster described indicate production generated from social psychology as well as from other disciplines such as anthropology or sociology. The life stories in this case can be oriented both to the implication of the context in the conception that the person makes of himself and to the knowledge of the group or society to which the person interviewed belongs.

#### Co-citation analysis

Co-citation of authors refers to the phenomenon by which two or more authors are cited together. When this occurs with significant frequency, it can be assumed that these authors work in the same area of knowledge; however, they are not necessarily collaborating (Ardanuy, 2012). The co-citation links show the relationships between two authors, and the color of the nodes distinguishes the group to which each author is associated by their thematic similarity (Galvez, 2018). The benefit of using co-citation analysis is that, in addition to finding the most influential publications, scholars can also discover thematic clusters (Donthu et al., 2021) to join. The largest nodes are linked to the authors who receive a high number of citations.

**FIGURE 4:**
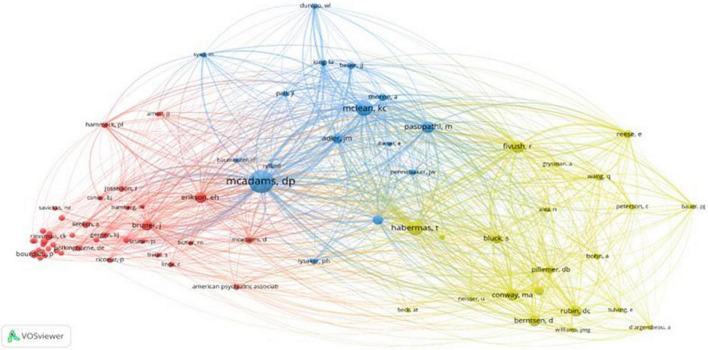
Co-citation of authors.

Figure 4 shows that there are three major schools of thought within the field of life stories. They are led by the main producers of knowledge about this technique (Table 4).

**TABLE 4:** Main producers of each school of knowledge.

Author	Occurrence	Author	Occurrence	Author	Occurrence
**Personality and identity**		**Autobiographical memory**		**Society and culture**	
D. P. McAdams	1,695	T. Habermas	681	U. H. Erikson	362
K. C. Mclean	746	R. Fivush	524	P. Bourdieu	222
M. Pasupathi	436	D. C. Rubin	398	C. K. Riessman	177
J. M. Adler	327	M. A. Conway	391	D. McAdams	160
H. A. Singer	295	D. Berntsen	386	E. Goffman	150
Thorne	204	J. Bruner	347	K. J. Gergen	137
J. J. Bauer	166	S. Bluck	294	P. Ricoeur	119
P. H. Lysaker	137	D. K. Thomsen	243	P. L. Hammack	116
Pennebaker	137	D. B. Pillemer	235	V. Braun	115
L. A. King	131	E. Reese	211	M. Foucault	113

##### Blue cluster: Personality and narrative identity

It is the most central cluster. It groups together authors who investigate the development of personality and narrative identity throughout the life cycle (McAdams, 2015). Among the authors with greater prominence within this cluster is McAdams, the main producer of knowledge about life stories. McLean, the second most prolific author, focuses his work on the development of narrative identity among adolescence and emerging adults, similar to the studies developed by Pasupathi. There is a slight transition toward the themes that compose the red cluster due to the importance of context in the development of personality because it is constructed through social interactions and genetics (McAdams, 1996; Barlow, 2019).

##### Yellow cluster: Autobiographical memory

In this cluster, we find the presence of very prolific authors such as Habermas, Fivush, and Bluck. These authors analyse the construction of autobiographical memory as well as the way in which its development occurs (Fivush et al., 2011). Likewise, they address the stability of memory as a function of age or life events (Köber and Habermas, 2017; Camia and Habermas, 2020).

##### Red cluster: Culture and society

This is the most peripheral cluster. As the name indicates, it incorporates the social and cultural dimensions of studies conducted through life stories. This group includes Erikson, Bruner, and Hammack as the main references, in addition to great references in psychology, such as Freud, the father of psychoanalysis, or Vygotsky, the founder of historical-cultural psychology. This cluster also includes contemporary sociologists such as Glen Elder Catherine, Kohler Reissman, and Gabriele Rosenthal, and great names in this discipline, such as Pierre Bourdieu, Michel Foucault, Anthony Giddens, and Erving Goffman.

The graphic analysis again shows that the accounts created doctrine within psychology because most of the authors cited at length are psychologists. However, in the literature referenced in the red cluster, eminent sociologists stand out, although their lower relative prevalence suggests that they compose more the theoretical basis of the sociocultural approach than a source of empirical research.

#### Document co-citation analysis

Co-citation analysis occurs when two elements of the literature are cited together by a third party (Miguel et al., 2007). In addition, if it is assumed that a highly cited documents represent key concepts, methods, or experiments in a certain field, co-citation can be used to identify and visualize the relationship between these key ideas (Small, 2013). Co-citation analysis is a technique for scientific mapping that assumes that publications that are frequently cited together are thematically similar (Hjørland, 2013). In this sense, co-citation analysis has proven to be a useful empirical technique to describe the intellectual structure of the disciplines (Miguel et al., 2007; Sainaghi et al., 2018).

**FIGURE 5:**
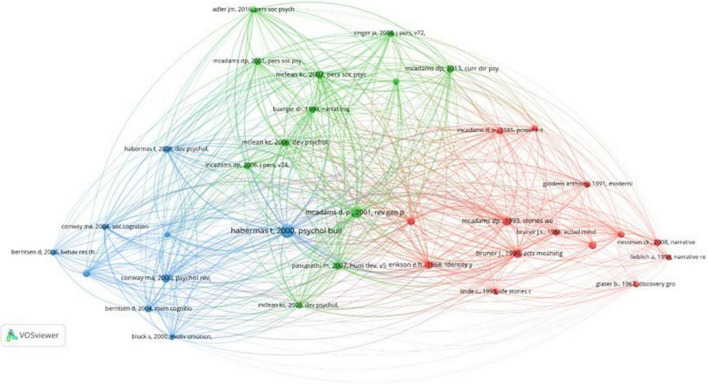
Co-citation of documents.

Figure 5 shows three clusters that represent the intellectual structure developed around life stories. Each of these three clusters identifies and visualizes the works that address the different topics of the field of study.

##### Green cluster: Personality and narrative identity

This is the most central cluster and includes the work of the main producers of knowledge. We find works by McAdams et al. (2001), McAdams and Pals (2006), McLean and Pratt (2006), McLean et al. (2007), Pasupathi et al. (2007), McAdams (2013), and Adler et al. (2016). These works deal with the study of narrative identity from different approaches; however, the psychosocial impact on narrative identity throughout the lives of people is commonly identified.

##### Blue cluster: Autobiographical memory

The main works in this cluster belong to authors such as Bluck and Habermas (2000), Conway and Pleydell-Pearce (2000), Habermas and Bluck (2000), Berntsen and Rubin (2004), or Conway et al. (2004). Their work deals with the concept of autobiographical memory as such. The authors work on this term as the union not only of individual events but also of life and its coherence, giving rise to a diagram of the life history of people.

##### Red cluster: Culture and society

This cluster includes the work developed by authors such as Kemph (1969), Bruner (1990), McAdams et al. (1993), and Habermas and Bluck (2000) pertaining to the search and construction of identity and how it is influenced by a cultural system. It also includes the work of Riessman (2008), who offers guidance in terms of social research, specifically the use of narrative methods. In this sense, the contributions of authors such as McAdams show the closeness between the two clusters and the intellectual influences of the same author in different fields of knowledge.

The graph again shows a very connected network, revealing the existence of deep intellectual influences between the different works. There are no large central works but many and very connected ones. Therefore, the knowledge is based on numerous and current research. This distribution, again, is a trend that is typical of experimental techniques. However, once again, this trend is nuanced in the red cluster, where the presence of older works indicates the influence of trends more typical of the humanities, for which research is based on previous works to support studies.

## Discussion

This study has shown the important role that literature reviews play in integrating and synthesizing existing knowledge and providing state-of-the-art understanding, identifying gaps, and inconsistencies in the literature, as well as pointing to avenues for future research to address outstanding issues and advance knowledge (Paul et al., 2021; Lim et al., 2022).

The work developed herein is relevant because it allows us to shed light on this matter. Through this first bibliometric analysis of the scientific production generated through life stories, we have gained knowledge of one of its techniques.

All the results indicate that we are facing a strongly consolidated technique. With almost a century of use, life stories have gone through different stages, reaching maturity recently. However, based on the analysis of the body of articles, we conclude that such maturity occurs in the field of psychology. We did not find references among the most prominent researchers to those who approach their studies from fields such as sociology, anthropology, or social work, which displaces their work to a more residual position. However, it is possible that the knowledge produced by sociologists or anthropologists will be transferred in other spaces and in other forms (Delgado-Baena et al., 2022).

Likewise, when analyzing the journals in which these researchers publish, there is a diversity of categories. However, among them, those belonging to the field of psychology are once again taking center stage. There are four topics addressed by these publications: personality and narrative identity, autobiographical memory, health and society, and culture. The first two present a clear psychological approach. The third is related to production developed from the health sciences. Only in the fourth category is there a contribution to social problems from sociology or anthropology. However, using bibliometric analysis, we cannot distinguish the weight or perspective of these disciplines because their relational techniques do not allow us to deepen this knowledge.

These topics are addressed by three major schools of thought personality and narrative identity, autobiographical memory, and society and culture, which are led by the main producers of knowledge. Clear cooperation occurs between them, leading to the fact that in these schools, knowledge is based on numerous and current investigations that are discussed, which again shows trends typical of the experimental techniques.

From the analyses completed thus far, we can conclude that the production developed from the field of psychology occupies a privileged position in terms of the production and transmission of knowledge through life stories. In contrast, the contributions that are made from sociology or anthropology in this field are blurred, relegating to the background the work carried out by its scholars. The technique of life history in the scientific field is thus associated with the psychological, being concerned mainly with the development of the personality in its relationship with the social or cultural environment, rather than with the social facts themselves.

The displacement of work from these disciplines is due, above all, to external causes and not intrinsic weaknesses of the specialties themselves. The monopoly of science means that those disciplines with more empiricist approaches, such as psychology, have a greater place in the science market. Meanwhile, other types of contributions do not leave a mark on the scientific literature, blurring the information in the databases, which also do not collect all the production that is developed (Moravcsik, 1989). Similarly, the analysis concentrates only on highly cited publications, and leaves publications that are recent or niche outside their subject groups (Donthu et al., 2021).

The hegemony of psychology over sociology or anthropology can reduce the other forms of observation and theorization to a marginal, precarious existence, or ultimately to their disappearance (Bertaux, 1980).

In this sense, a contribution of this study, in addition to helping researchers and readers understand the general intellectual structure of the field of life stories, is the identification of the need for future research that delves into the disciplinary differences in the use of this technique. Continuing systematic reviews of the literature produced through the other techniques of the biographical method and the triangulation of these studies is a matter of urgency for the optimal development of this method, as well as the advancement of scientific knowledge in this field.

Likewise, the results demonstrate to research communities, government institutions, and funding agencies the need to value scientific spaces with more constructivist views on social problems, such as sociology or anthropology, as well as develop solutions from the perspective of these disciplines.

## Data availability statement

The original contributions presented in this study are included in the article/supplementary material, further inquiries can be directed to the corresponding authors.

## Author contributions

RL-M, CG-N, and AS: conceptualization, methodology, and formal analysis. RL-M: data curation and writing—original draft preparation. CG-N and AS: writing—review and editing and supervision. RL-M, CG-N, AD-B, RV-J, and AS: validation, investigation, resources, visualization, read, and agreed to the published version of the manuscript.
